# Study of independent diagnostic efficacy and co-diagnostic strategies of molecular markers for diabetic cardiomyopathy

**DOI:** 10.3389/fendo.2025.1618230

**Published:** 2025-08-12

**Authors:** Na Su, Jingxuan Zhao, Weiyi Zhang, Xinhuan Zhang, Kunna Lu, Yan Ma, Yan Wang, Mingfeng Cao

**Affiliations:** ^1^ Graduate School, Shandong First Medical University & Shandong Academy of Medical Sciences, Jinan, China; ^2^ Department of Endocrinology, Second Affiliated Hospital of Shandong First Medical University, Tai’an, China

**Keywords:** diabetic cardiomyopathy, molecular markers, oxidative stress, non-coding RNA, early diagnosis

## Abstract

Word count of the full article: 4834Diabetic cardiomyopathy (DCM) is defined as myocardial dysfunction in diabetes mellitus (DM) patients independent of coronary artery disease (CAD) or hypertension (HTN). With high morbidity and mortality, DCM poses a significant threat to patient health. Its underlying pathogenesis remains incompletely elucidated, and the prolonged subclinical phase renders early diagnosis and precise treatment clinically challenging. Thus, identifying viable biomarkers for early diagnosis and intervention has emerged as a research imperative, whereas a systematic DCM diagnostic and therapeutic strategy remains to be established. Our examination revealed that circulating soluble suppression of tumorigenicity 2 (sST2), Cardiotrophin-1 (CT-1), and galectin-3 levels correlate closely with DCM progression stages. Combining Lysyl Oxidase-Like 2 (LOXL2) and Electron Transfer Flavoprotein β Subunit (ETFβ) measurements with ultrasound E/E’ ratio and NT-proBNP enhances diagnostic accuracy. Novel noninvasive markers (e.g., skin autofluorescence) show promise. This article comprehensively evaluates the clinical applications of these molecular markers within DCM’s pathophysiological classification framework.

## 1 Introduction

DM is a global public health problem, affecting 4%–17% of the population worldwide. The International Diabetes Federation (IDF) predicts that the number of DM patients will exceed 610 million by 2045 (1). Cardiovascular complications of DM, such as myocardial remodeling and heart failure (HF)—account for 80% of DM-related deaths. DM is an independent predictor of HF, even after excluding traditional risk factors like CAD and HTN. In 2017, Lorenzo-Almorós et al. (2) proposed the first diagnostic criteria for DCM, with core features including left ventricular diastolic dysfunction (LVDD), reduced ejection fraction (EF), pathological myocardial hypertrophy, and interstitial fibrosis. Although recent studies have elucidated DCM’s pathological mechanisms (e.g., abnormal myocardial metabolism, mitochondrial dysfunction, oxidative stress, and fibrotic cascade activation) (3), early diagnosis remains challenging due to nonspecific cardiac alterations and a prolonged subclinical phase. This review systematically integrates the latest research progress on novel molecular markers (e.g., soluble suppression of tumorigenicity 2 [sST2], adiponectin [APN], and develops strategies for “molecular marker combinations” and “integration of molecular markers with imaging technologies” to provide a theoretical basis for the early and accurate diagnosis of DCM.

## 2 Indicators associated with myocardial oxidative stress and inflammatory response

### 2.1 soluble suppression of tumorigenicity 2

Suppression of tumorigenicity 2 (ST2), a member of the interleukin-1 receptor family (IL-1R family), exists in two subtypes: transmembrane (ST2L) and soluble (sST2). As a decoy receptor for IL-33, sST2 competitively binds to IL-33, thereby interfering with the IL-33/ST2L signaling pathway and modulating pathological processes such as atherosclerosis and cardiac remodeling (4). Fousteris et al. (5) demonstrated that plasma sST2 levels were significantly elevated in patients with T2DM compared to healthy controls, with further increases observed in those with comorbid left ventricular diastolic dysfunction (LVDD). While existing studies have established the prognostic value of sST2 in heart failure with mid-range ejection fraction (HFmrEF) (6), its predictive efficacy in HFmrEF patients with comorbid T2DM remains unclear.

### 2.2 Nod-like receptor family pyrin domain containing 3 inflammasome (NLRP3 inflammasome)

The Nod-like Receptor Family Pyrin Domain Containing 3 Inflammasome (NLRP3 inflammasome) is a multimeric complex consisting of NLRP3 protein and apoptosis-associated speck-like protein (ASC). This multimeric complex, whose aberrant activation may be closely associated with the pathological processes of autoimmune and metabolic diseases. Luo et al. (7) found that high glucose-induced oxidative stress activates the NLRP3 inflammasome via thioredoxin-interacting proteins, thereby promoting cardiomyocyte apoptosis and myocardial fibrosis. A 2020 review (8) summarized the dual role of NLRP3 in DCM: short-term anti-inflammatory benefits, but long-term overactivation exacerbates myocardial injury. Kai et al. (9) demonstrated that NLRP3 activation leads to the release of IL-1β and IL-18, which exacerbate myocardial fibrosis, oxidative damage, and disturbed energy metabolism; thus, targeting the NLRP3 inflammasome may serve as a therapeutic strategy for DCM.

### 2.3 Growth differentiation factor-15

Growth differentiation factor-15 (GDF-15), a member of the transforming growth factor-β (TGF-β) superfamily, plays a key role in regulating inflammatory responses and promoting cell growth and differentiation. Notably, Dominguez-Rodriguez et al. (10) reported that serum GDF-15 levels were significantly upregulated in asymptomatic DCM patients. Additionally, a review highlighted that high glucose induces GDF-15 expression in endothelial cells via the p53 pathway, which subsequently attenuates endothelial cell apoptosis by activating the PI3K/Akt/eNOS pathway, thus suggesting GDF-15’s potential role in DM-related vascular protection (11). However, due to the lack of specificity of GDF-15 in metabolic diseases (12), its clinical utility in predicting disease progression remains controversial.

### 2.4 High mobility group A1 and high-mobility group box 1

High mobility group A1 (HMGA1), a specific cofactor for gene activation, plays a critical role in key biological processes, including cell cycle regulation, embryonic development, tumor cell transformation and differentiation, apoptosis, and DNA repair. In cardiac muscle and fibroblasts of DM mice, HMGA1 expression is significantly elevated, and its overexpression promotes autophagy through the miR-222-mediated P27/CDK2/mTOR signaling pathway, which in turn exacerbates inflammatory responses and apoptosis in cardiomyocytes in a high-glucose environment, and participates in the process of cardiac remodeling in DCM (13).

High-mobility group box 1 (HMGB1) is a highly conserved nuclear protein commonly found in various tissues and cells of mammals, and plays an important role in the regulation of inflammatory responses. Tao et al. (14) found that cardiomyocyte-derived HMGB1 promoted DCM by inhibiting fibroblastic IL-33 expression via TLR4 and promoting collagen production. Both resveratrol and ustekinumab induced the downregulation of HMGB1 expression, demonstrating a protective effect against DCM: Resveratrol prevented oxidative damage, myocardial fibrosis, and inflammation by inhibiting the HMGB1/RAGE/TLR4/NF-κB signaling pathway (15), whereas ustekin exerted a protective effect against DCM by reducing inflammation and cardiomyocyte apoptosis through inhibition of the JNK and p38 signaling pathways (16).

### 2.5 Chitinase-3-like protein 1

Chitinase-3-like protein 1 (CHI3L1/YKL-40) belongs to the chitinase family and is involved in endothelial dysfunction and tissue remodeling. CHI3L1/YKL-40 is highly expressed in a variety of infectious and non-infectious inflammatory diseases and is considered a noninvasive prognostic biomarker of inflammation. Serum CHI3L1/YKL-40 levels in patients with DCM were positively correlated with left ventricular mass index (LVMI)and degree of LVDD (17). In a multicenter cohort study, it was observed that CHI3L1/YKL-40 was significantly elevated in the serum of patients with DCM, and its levels correlated with markers of myocardial fibrosis (e.g., PIIINP) and myocardial scar area as detected via Cardiac Magnetic Resonance Imaging (CMR) (18).

### 2.6 Neutrophil-to-lymphocyte ratio

Neutrophil-to-lymphocyte ratio (NLR) is a simple and cost-effective index for assessing inflammatory status, which is widely used in clinical and scientific research. Huang et al. (19) found that serum NLR was significantly higher in the DCM group than in the T2DM-only group in a cross-sectional study of 507 T2DM patients, suggesting that NLR is associated with subclinical DCM.

### 2.7 C-reactive protein

C-reactive protein (CRP) is a plasma protein that participates in the systemic inflammatory response as an acute-phase reactant, and its plasma concentration is elevated in the inflammatory state. The high-sensitivity C-reactive protein (hsCRP) assay can accurately measure low concentrations of CRP in blood. A study by Mano et al. (20) found that elevated CRP levels exacerbated LVDD in patients with DCM, a finding that is closely related to myocardial inflammatory responses, angiotensinogen levels, and AT1 receptor expression.

### 2.8 Other pro-inflammatory cytokines

During the pathological process of DM, cardiomyocytes and fibroblasts release a variety of pro-inflammatory cytokines, including IL-1, IL-6, IL-12, IL-18, and TNF-α, which mediate the pathological damage induced by glycotoxicity and lipotoxicity by triggering mitochondrial dysfunction, oxidative stress, insulin resistance (IR), and β-cell apoptosis and other pathways. Epidemiologic studies conducted in areas with a high prevalence of DM (e.g., West Virginia, USA) (21) have shown that serum TNF-α, IL-6, and isoprostanes (IsoPs) levels are significantly elevated in patients with early-stage DCM, whereas bilirubin (Bil) concentrations are decreased. Although plasma concentrations of proinflammatory cytokines alone cannot distinguish DCM from other myocardial diseases, their dynamics have been shown to assist in assessing the pathologic progression of DCM.

## 3 Indicators of myocardial metabolic disorders

### 3.1 Adiponectin

Adiponectin (APN) is an adipokine composed of 244 amino acids and belongs to the collagen superfamily. It is involved in the regulation of glucolipid metabolism by binding to its cognate receptors, promoting fatty acid oxidation and glucose uptake, and enhancing insulin (INS) sensitivity. Shaver et al. (21) found that serum levels of APN were lower than those in normal controls in patients with DM and DCM, and this reduction was particularly significant and were particularly significant in patients with DCM. In a rat model of DCM (22), reduced myocardial and serum levels of APN were negatively correlated with IR index, triglyceride (TG) and total cholesterol levels, suggesting that APN may influence the progression of DCM through IR. In addition, Battiprolu et al. (23) demonstrated that APN antagonized the progression of DCM by reducing cardiomyocyte death by inhibiting cardiomyocyte hypertrophy and inflammatory responses.

### 3.2 Insulin-like growth factor binding protein-7

Insulin-like growth factor binding protein-7 (IGFBP-7), a member of the IGFBP superfamily, regulates the insulin (INS) receptor signaling pathway by binding to insulin-like growth factor-1. The results of Shaver et al. (21) showed that plasma levels of IGFBP-7 were significantly increased in patients with DCM. Ruan et al. (24) demonstrated that higher levels of IGFBP-7 were associated with worse clinical characteristics and increased risk of adverse clinical outcomes in patients with HFrEF. All of the above suggests that IGFBP-7 may be a biomarker for early diagnosis of DCM and cardiac fibrosis.

### 3.3 Activin A

Activin A, a member of the TGF-β superfamily, is secreted by epicardial adipose tissue. Greulich et al. (25) found that activin A signaling overactivation led to IR and cardiac systolic dysfunction in a guinea pig model induced by STZ/HFD. Further studies showed that plasma activin A levels were significantly elevated in T2DM patients with impaired myocardial glucose metabolism and left ventricular remodeling compared with those with T2DM alone (26). Molecular mechanism studies have shown that activin A exacerbates myocardial metabolic disturbances by inhibiting INS-mediated phosphorylation in the PI3K/Akt signaling pathway, which is a key regulatory pathway for myocardial glucose uptake, revealing its central role in the pathological process of DCM (27).

### 3.4 Other indicators of metabolic disorders

A prospective study in the United Kingdom (28) showed a significant positive correlation between the incidence of HF and the level of glycated hemoglobin (HbA1c) in patients with T2DM, highlighting the clinical value of HbA1c as an indicator of long-term glycemic control. Advanced glycation end products (AGEs) are heterogeneous macromolecules formed by cross-linking sugar molecules with proteins, lipids, and nucleic acids through non-enzymatic reactions. Accumulation of AGEs in a high-glycemic environment induces cross-linking of myocardial collagen and other proteins, triggering interstitial fibrosis and ultimately leading to LVDD (29). Leptin, a circulating signaling molecule secreted by white adipocytes, mainly reflects TG storage status and is involved in the regulation of obesity and related metabolic disorders. Shaver et al. (21) found that serum leptin levels were significantly elevated in patients with DM combined with LVDD (especially in the high BMI group) and negatively correlated with APN levels, suggesting that leptin may become an early biomarker in DCM patients with high BMI. IsoPs are specific products of peroxidation of polyunsaturated fatty acids, and Luo et al. (30) found that hyperoxidative stress in myocardial tissues was accompanied by a significant increase in IsoPs levels in a T1DM rat model. Bilirubin (Bil), as a heme catabolic metabolite, has antioxidant properties since its antioxidant properties were first demonstrated by Ames et al. in 1987, and Chung et al. (31) found a negative correlation between serum total bilirubin levels and the incidence of cardiovascular autonomic neuropathy by analyzing data from 3015 T2DM patients.

## 4 Myocardial fibrosis-related indicators

### 4.1 Galectin-3

Galectin-3, a member of the lectin family, is involved in the regulation of fibrotic and inflammatory responses in cardiac, renal, and hepatic diseases. Flores-Ramírez et al. (32) demonstrated that the serum concentration of Galectin-3 was significantly elevated in patients with DM combined with reduced LVEF, suggesting that it may be a potential biomarker for identifying the early stages of LVDD in DM patients. In a 2022 review (33), Galectin-3 was described as a potential therapeutic target in DCM, where it may exacerbate cardiac dysfunction by promoting myocardial fibrosis and inflammatory responses.

### 4.2 Cardiotrophin-1

Cardiotrophin-1 (CT1), a member of the gp130 family, is a potent inducer of cardiomyocyte hypertrophy. Gamella-Pozuelo et al. (34) found that serum CT-1 levels were significantly elevated in patients with T2DM and HTN and were positively correlated with LV hypertrophy and arterial stiffness. Gamella-Pozuelo et al. (35) demonstrated a significant positive correlation between plasma CT-1 levels and basal blood glucose levels and the degree of left ventricular hypertrophy in patients with T2DM, and CT-1 concentrations were significantly higher in individuals with impaired glucose tolerance or newly diagnosed DM than in healthy controls.

### 4.3 Transforming growth factor-beta

Transforming Growth Factor-beta (TGF-β), as a member of the multifunctional cytokine superfamily, plays a central regulatory role in cell proliferation, differentiation, migration, and the dynamic homeostasis of the extracellular matrix (ECM).A 2017 review (36) indicated that TGF-β can promote myocardial fibroblast proliferation and collagen deposition through Smad-dependent and Smad-independent pathways and inhibit matrix metalloproteinase (MMP) activity, which in turn leads to myocardial fibrosis and ventricular remodeling; whereas TGF-β-neutralizing antibodies attenuates myocardial collagen deposition and improves diastolic dysfunction in DM mice.

### 4.4 N-terminal propeptide of procollagen type I and N-terminal propeptide of procollagen type III

Collagen Type I and Collagen Type III are the most abundant collagen types in the myocardium and other tissues, and serum N-terminal Propeptide of Procollagen Type I (PINP) and N-terminal Propeptide of Procollagen Type III (PIIINP) serve as specific biomarkers for their synthesis, respectively. Among them, PIIINP levels can also be used to assess the status of ECM renewal. Ihm et al. (37) showed that serum PINP was significantly elevated in patients with early-stage T2DM, which was significantly associated with myocardial fibrosis and LVDD. Quilliot et al. (38) found that PIIINP levels were significantly associated with early-stage LVDD in IR individuals in a normotensive, non-DM population.

### 4.5 Cellular communication network factor 2

Cellular communication network factor 2 (CCN2/CTGF) is a cysteine-rich stromal cell protein that plays an important role in connective tissue growth regulation and angiogenesis. Studies have shown that CCN2/CTGF induces cardiomyocyte hypertrophy and apoptosis during the pathological process of DCM, and CCN2/CTGF may serve as a sensitive biomarker for the early diagnosis of DCM and potentially play a protective role in myocardial injury (39).

## 5 Indicators related to myocardial steatosis and other injuries

### 5.1 Fatty acid binding proteins

Fatty Acid Binding Proteins (FABPs), as lipid chaperone proteins, are involved in multi-organ metabolic networks through the regulation of lipid flow, transport, and signaling. The STZ/HFD-induced T2DM mouse model showed that serum FABP4 levels were elevated in parallel with myocardial neutral lipid content, suggesting that it may be a novel biomarker for assessing myocardial lipid deposition (40). Human heart-type fatty acid binding protein (H-FABP), a myocardial-specific cytoplasmic protein responsible for transporting fatty acids to mitochondria for oxidative energy supply, is usually not detected in the serum of healthy individuals. Notably, Akbal et al. (41) found that serum H-FABP levels were significantly elevated in patients with T2DM combined with early cardiac injury. Taken together, the FABP family (especially FABP4 and H-FABP) may serve as a potential biomarker combination for the early diagnosis of DCM.

### 5.2 Natriuretic peptide family

Atrial natriuretic peptide (ANP) and brain natriuretic peptide (BNP), as cardiac endocrine hormones, act through activating the guanylate cyclase (GC)/cGMP signaling pathway to exert vasodilatory, natriuretic (sodium-excretory), and kaliuretic (potassium-excretory) effects, while inhibiting the renin-angiotensin-aldosterone system (RAAS) and sympathetic activity. In the STZ-induced DM rat model, BNP was compensatorily elevated in plasma and atrial myocardial tissue, accompanied by a decrease in short-axis shortening of cardiomyocytes, suggesting that it may delay the deterioration of cardiac function through a negative feedback mechanism (42). Mid-range atrial natriuretic peptide precursor (MR-proANP) is a mid-range fragment of ANP, and Jensen et al. (43) found that MR-proANP was positively correlated with left atrial volume and LVMI in patients with T2DM complicated with Heart Failure with Preserved Ejection Fraction (HFpEF), this finding provides a new tool for accurate stratification of HFpEF.

N-terminal Pro-Brain Natriuretic Peptide (NT-proBNP), an inactive precursor fragment of BNP, has demonstrated clinical value in the diagnosis of HF superior to BNP because of its long plasma half-life and stability. Malachias et al. (44) found that elevated NT-proBNP levels were significantly and positively associated with all-cause mortality, cardiovascular mortality, and HF in patients with T2DM. The Portuguese Multi-Society Consensus (45) was the first to systematically standardize the clinical use of NT-proBNP in patients with DM, emphasizing routine testing in people over 50 years of age, initiating screening in younger patients based on risk factors, and recommending individualized adjustment of thresholds to provide a practical framework for the early identification of cardiac lesions and the development of therapeutic regimens.

### 5.3 Fibroblast growth factors

Fibroblast Growth Factors (FGFs), a superfamily of peptide growth factors, are involved in embryonic development, organogenesis, and maintenance of metabolic homeostasis by activating FGF receptor tyrosine kinase activity. FGF-21 is mainly secreted into the bloodstream by the liver, but cardiomyocytes can also synthesize it autocrinally. In the STZ/HFD-induced DM mouse model (46), FGF-21 knockout mice showed a significant increase in myocardial lipid uptake and deposition, and Yang et al. (47) found that FGF21 inhibited oxidative stress and lipid accumulation through the AMPK/AKT/Nrf2 pathway to improve cardiac function in T2DM mice, which provides a new target for DCM intervention. Sørensen et al. (48) analyzed 246 T2DM patients by CMR and found that FGF-23 levels were significantly associated with LVDD and reduced myocardial perfusion. In conclusion, FGF-21/FGF-23 may be a novel molecular marker for the diagnosis and prognosis of DCM.

### 5.4 Myocardial TG and their intermediate metabolites

In patients with DM, abnormal glucose uptake and hypermetabolism of fatty acids (FAs) lead to abnormal accumulation of myocardial TG and cholesterol. Rijzewijk et al. (49) found that myocardial TG deposition in patients with uncomplicated T2DM was significantly positively correlated with LVDD, and that this correlation was independent of age and BMI. Abnormal accumulation of cardiac TG can produce a toxic metabolic intermediate, ceramide (Cer). Cer protects cell membranes from damage by free FAs, but in excess it induces apoptosis and fibrosis. Studies have shown that Cer levels can independently predict the risk of T2DM and cardiovascular disease (50), but its clinical value as a diagnostic marker for DCM needs further validation.

### 5.5 G protein-coupled receptor kinase-2

G protein-coupled receptor kinase-2 (GRK2) belongs to the serine/threonine family of protein kinases and is involved in myocardial contraction, angiogenesis, inflammation, and metabolic homeostasis by phosphorylating and regulating the desensitization and internalization of G protein-coupled receptors (GPCRs). Lai et al. (51) observed significant upregulation of GRK2 expression in both myocardial tissue and peripheral blood mononuclear cells in a mouse model of DCM and in DM patients complicated with LVDD. These findings suggest that GRK2 may be a novel molecular marker for the early diagnosis of DCM.

### 5.6 3-Nitrotyrosine

3-Nitrotyrosine (3-NT), a nitration product of protein tyrosine residues, induces structural and functional changes in proteins and mediates myocardial mitochondrial impairment. STZ-induced T2DM rat model showed a positive correlation between the expression of 3-NT and the number of apoptotic myocytes in myocardial tissues, and treatment with valsartan significantly reversed 3-NT elevation and apoptotic cell death in the diabetic state, suggesting its potential as a diagnostic and therapeutic efficacy assessment marker (52). Jakubiak et al. (53) systematically reviewed the role of 3-NT in diabetic cardiovascular complications, emphasizing its association with myocardial nitrative stress, fibrosis, and diastolic dysfunction, and suggesting that future focus should be on the function of nitration-modified proteins.

## 6 Non-coding RNA

Non-coding RNA (ncRNA), as an important gene regulatory molecule, is involved in myocardial fibrosis, abnormal energy metabolism, and other pathological processes in DCM through epigenetic and post-transcriptional regulation. A review published by our team in 2024 (54) systematically summarized the research progress of ncRNA in the early diagnosis of DCM, revealing that it can be used as a biomarker reflecting myocardial remodeling and a therapeutic target for intervening in the fibrotic pathway. Future studies will further explore the potential of ncRNA as a molecular marker for DCM.

## 7 Novel molecular markers

### 7.1 Endothelin 1

Endothelin 1 (EDN1) is a vasoactive peptide encoded by the EDN1 gene, which exerts biological effects such as vasoconstriction, proliferation/migration-promoting activity, and inflammatory activation by binding to endothelin receptor A/B (ETA/ETB). Hyperglycemia and metabolic disorders are involved in the pathology of DCM through activation of the immune system. Widyantoro et al. (55) showed that knockdown of the EDN1 gene in a mouse model of DM could reverse myocardial fibrosis and improve cardiac function. Guo et al. (56) found that the expression of the EDN1 gene was significantly upregulated in the myocardial tissues of patients with DCM, and the expression level was positively correlated with the degree of macrophage infiltration. Mechanistic studies showed that EDN1 may promote myocardial inflammation by activating the NF-κB pathway, suggesting that EDN1 may serve as a potential biomarker of the immune microenvironment in DCM.

### 7.2 Skin autofluorescence

As previously mentioned, AGEs have been demonstrated to possess potential as biomarkers for DCM. Skin autofluorescence (SAF), as a non-invasive biomarker reflecting tissue deposition of AGEs, can be quantitatively measured via a dedicated SAF reader. By leveraging the inherent fluorescent properties of AGEs, SAF intensity correlates with their accumulation and facilitates the assessment of disease progression. A Dutch cohort study (57) showed that SAF values were independently associated with cardiovascular risk (CVD) and all-cause mortality in patients with T2DM, with a 2.59-fold increase in the risk of CVD events or death for every 1-unit increase in SAF. A Japanese cross-sectional study (58) found that SAF values were significantly and positively correlated with high-sensitivity cardiac troponin T (hs-cTnT) levels in DM patients. As a non-invasive test, SAF may become a novel tool to replace invasive tests for early identification of DCM.

## 8 Combined diagnosis

Currently, the combined application of molecular markers has become an important strategy to improve the accuracy of disease diagnosis. By integrating the combined detection of molecular markers and the combined analysis of molecular marker-imaging technique combinations, not only can the molecular pathological mechanisms of the disease be more comprehensively analyzed, but also the diagnostic sensitivity and specificity can be significantly improved, thus enabling early and accurate identification and typing of the disease. The following section describes the potential applications of several molecular markers and their combination with imaging techniques in the diagnosis of DCM, and their key roles in enhancing diagnostic efficacy (see **Table 1** for details).

**Table 1:** Relevant literature and efficacy analysis of biomarker combined diagnosis.

Type of study	Study group and subjects	Detection indicators	Results	Author, country, year.
Animal experimental study	T2DM group (db/db mice); N = 40; Age: 6–16 weeks Control group (db/+ mice); N = 40; Age: 6–16 weeks	LOXL2 and ETFβ	In db/db mice, the combined detection of elevated serum LOXL2 levels and decreased serum ETFβ levels demonstrated superior predictive efficacy (AUC = 0.813), outperforming single indicators alone.	Johnson et al. (59), South Africa, 2020
Review	None	ANGPTL4 and LPL	Maintaining the balance of LPL and ANGPTL4 levels is crucial for preventing DCM lesions, and their combined detection helps in early problem identification.	Puthanveetil et al. (60), Canada, 2015
Cross-sectional study	T2DM group; N = 78; age: 45–65 yr Control group; N = 12; age: 45–65 yr	miR-1, miR-133a	1. The levels of miR-1 and miR-133a were positively correlated with myocardial fat deposition (*r* = 0.68, *p* < 0.001). 2. The diagnostic efficacy (AUC = 0.89) of miR-1/miR-133a combined with HbA1c for myocardial fat deposition was significantly higher than that of the single-index.	De Gonzalo - Calvo et al. (61), Spain, 2016
Cross-sectional study	DCM group; N = 49; age: 56.33 ± 8.19 yr; T2DM group; N = 49; age: 53.35 ± 8.29 yr	miR-21, HbA1c, DM duration, and blood lipid levels	Combining miR-21, DM duration, HbA1c%, and blood lipid indices achieves the highest diagnostic efficiency (AUC = 0.939).	Tao et al. (62), China, 2000
Cross-sectional study	DCM group; N = 92; age: 60.18 ± 12.26 yr T2DM group; N = 105; age: 57.50 ± 15.02 yr	Butyric acid and the methylation status of 7 CpG sites in intron 1 of HIF3A	The AUC of combining butyric acid with CpG-6 methylation reached 0.737, which was superior to that of the single-index.	Guo et al. (63), China, 2021
Cross-sectional study	T2DM group; N = 60; age: 45–65 yr Control group; N = 40; age: 45–65 yr	MMPs and TIMPs	In patients with DM complicated with LVDD, the levels of MMP-9 and MMP-7 in plasma were elevated, and the ratio of TIMP-1 to MMP-9 was decreased.	Ban et al. (64), Australia, 2010
Cross-sectional study	DM-DD group; N = 47; age: 45–65 yr Simple DM group; N = 34; age: 45–65 yr	TNF-α, INS, AGEs, Cr	1.INS ≥22.7, TNF-α ≥3.9, AGEs ≥11.6, and Cr ≥1.1 are important predictive factors for distinguishing DM-DD from DM-N. 2.Combined markers (TNF-α + INS + AGEs + Cr) exhibit an AUC of 0.913 and a specificity of 100%.	Abdelrahman et al. (65), Egypt, 2021
Cross-sectional study	DM-DD group; N = 47; age: 45–65 yr DM-SD group; N = 32; age: 45–65 yr	IL-6, AGEs	1. AGEs ≥14.2 and IL-6 ≥6.4 are important predictive factors for distinguishing DM-SD from DM-DD. 2.Combined markers (IL-6 + AGEs) exhibit an AUC of 0.796 and a sensitivity of 90.6%.	Abdelrahman et al. (65), Egypt, 2021
Cross-sectional study	DCM (DM-DD + DM-SD) group; N = 79; age: 45–65 yr DM-N group; N = 34; age: 45–65 yr	TNF-α, IL-6, AGEs	1.TNF-α ≥1.7, AGEs ≥11.4, and IL-6 ≥3.5 are important predictive factors for distinguishing DCM from DM-N. 2. Combined markers (TNF-α + IL-6 + AGEs) exhibit an AUC of 0.924, a sensitivity of 84.8%, and a specificity of 88.2%.	Abdelrahman et al. (65), Egypt, 2021
Cohort study	DM-HF group; N = 385; age: 67.8 ± 10.3 yr Non-DM-HF group; N = 684; age: 65.3 ± 14 yr	sST2 and hs-cTnT	Combined detection of sST2 and hs-cTnT in plasma improves the diagnostic efficacy of clinical outcomes in HF patients with and without DM (AUC = 0.811) and is significantly superior to single markers.	Alonso et al. (66), Spain, 2016
Cohort study	DM group; N = 2266; age: average 57 yr Prediabetic group; N = 4533; age: average 57 yr	hs-cTnT, NT-proBNP, hs-CRP, LVH	Based on a simple integer score of hs-cTnT, NT-proBNP, hs-CRP, LVH, it can effectively stratify the heart failure risk in patients with diabetes and prediabetes.	Pandey et al. (67), USA, 2021
Cross-sectional study	DM-mdEF group; N = 14; age: 55.42 ± 8.51 yr DM-pEF group; N = 76; age: 55.25 ± 10.62 yr Control group; N = 31; age: 50.89 ± 9.66 yr	Gal-3 level, GLS	Galectin-3 > 2.71 ng/ml + GLS < -18%: The sensitivity for diagnosing LVDD is 85%, the specificity is 81%, and the negative predictive value (NPV) is 97%, suggesting that combined detection can effectively exclude low-risk populations.	Flores - Ramirez R et al. (68), Mexico, 2017
Cohort study	T1DM patients; N = 960; age: average 48 yr	NT-proBNP, echocardiographic index (E/e’)	Patients with NT-proBNP > 300 pg/mL and E/e’ > 12 have a cardiovascular risk incidence of 115 cases per 1000 person-years, which is 23 times that of the low-risk group (NT-proBNP < 150 pg/mL and E/e’ < 8).	Rørth et al. (69), Denmark, 2020

### 8.1 TNF-α, INS, AGEs and creatinine

Abdelrahman et al. (65) identified a set of biomarkers for detecting cardiac structural and functional changes in early-stage DCM. Among them, a combination of four biomarkers—TNF-α, INS, AGEs, and Creatinine (Cr)—could predict the occurrence of diastolic dysfunction (DD) in patients with T2DM, with a sensitivity of approximately 79% and specificity of 100%. Additionally, a two-biomarker combination (IL-6 and AGEs) differentiated between SD and DD in diabetic patients with a sensitivity of 90.6%. Meanwhile, a three-biomarker panel (TNF-α, IL-6, and AGEs) achieved approximately 85% sensitivity and specificity in distinguishing DCM patients from diabetic patients with normal cardiac function. These biomarkers may serve as predictors for early DCM diagnosis and could aid in developing strategies to prevent HF.

### 8.2 Lysyl oxidase-like 2 and electron transfer flavoprotein β subunit

Lysyl Oxidase-Like 2 (LOXL2), a key cross-linking enzyme of the LOX family, catalyzes the cross-linking of collagen to elastin in the ECM. LOXL2 activation via the TGF-β/Smad pathway in the diabetic state promotes myocardial fibroblast proliferation and collagen deposition. Zhao et al. (70) found that elevated serum LOXL2 levels promote cardiac fibrosis by enhancing collagen cross-linking, which in turn leads to cardiac contractile dysfunction and LVDD. Electron Transfer Flavoprotein β Subunit (ETFβ), as a key component of the electron transport chain, is involved in the oxidative energy supply of amino acids and fatty acids. Johnson et al. (59) found in a mouse model of DM that elevated serum LOXL2 levels accompanied by decreased levels of ETFβ were significantly correlated with a decrease in LVEF as detected by echocardiography, and that this alteration appeared earlier than the cardiac pathological structural changes.

### 8.3 Angiopoietin-like 4 and lipoprotein lipase

Angiopoietin-like 4 (ANGPTL4), a metabolic and vascular homeostasis regulatory protein, is involved in TG metabolism mainly through the inhibition of Lipoprotein Lipase (LPL) activity. In a DCM mouse model (71), ANGPTL4 gene expression was significantly upregulated and promoted cardiomyocyte apoptosis through the Focal Adhesion Kinase/Sirtuin 3/Reactive Oxygen Species signaling axis. In addition, the inhibitory effect of ANGPTL4 on LPL is closely associated with lipotoxicity and inflammatory responses in DCM (60), suggesting that maintaining the LPL-ANGPTL4 dynamic balance is critical for cardioprotection in DM.

### 8.4 MicroRNAs

De Gonzalo-Calvo et al. (61) found a dose-dependent positive correlation between myocardial fat deposition and serum miR-1/miR-133a levels in patients with T2DM and in a mouse model of IR. Tao et al. (62) demonstrated that blood miR-21 combined with HbA1c, the duration of DM and lipid parameters could significantly improve the early diagnosis of DCM by constructing a diagnostic model. These findings provide new evidence for the clinical translation of circulating microRNAs (miRNAs) as biomarkers for DCM.

### 8.5 Short-chain fatty acids and HIF3A mRNA

Short-chain fatty acids (SCFAs), which are metabolites of dietary fiber fermentation by colonic flora, mainly include acetic acid, propionic acid, and butyric acids. Guo et al. (63) first found that plasma levels of SCFAs were significantly lower in patients with DCM than in patients with T2DM alone, with the most significant difference in butyric acid (BA). The plasma BA level was negatively correlated with Cytosine-phosphate-Guanine-6 (CpG-6) methylation in a dose-dependent manner. This study suggests that the combination of plasma BA level and HIF3A intron 1 CpG-6 methylation status may be a novel combination of molecular markers to distinguish DCM from T2DM alone.

### 8.6 Matrix metalloproteinases and tissue inhibitors of metalloproteinases

Matrix Metalloproteinases (MMPs), a family of zinc-dependent endopeptidases, are involved in tissue remodeling by degrading ECM proteins. Cardiac fibroblasts maintain the dynamic homeostasis of the ECM through the secretion of MMPs and their inhibitors Tissue Inhibitors of Metalloproteinases (TIMPs). Ban et al. (64) observed an increase in plasma MMP-9/MMP-7 levels along with a decrease in the TIMP-1/MMP-9 ratio in patients with DM complicated with LVDD. These studies suggest that MMPs-TIMPs imbalance may serve as a novel combination of biomarkers for the assessment of diastolic dysfunction.

### 8.7 Cardiac troponin, NT-proBNP, hs-CRP, electrocardiogram

As a core regulator of myocardial contraction, the cTns consist of three subunits—cardiac troponin I (cTnI), cardiac troponin T (cTnT), and cardiac troponin C (cTnC)—among which cTnI and cTnT serve as highly sensitive markers of myocardial injury. hs-cTnT serves as a highly sensitive assay version of cTnT, enabling precise identification of small myocardial injuries, and several of the other markers have been mentioned above. In a prospective cohort study, Pandey et al. noted that a combined integer score of hs-cTnT, NT-proBNP, hs-CRP, and electrocardiogram (ECG)-diagnosed left ventricular hypertrophy (LVH, diagnosed by the Sokolow-Lyon criterion) was effective in stratifying the risk of HF in patients with DM and antecedent cardiovascular disease. and identifying those at high risk (scores of ≥3) (67), and that this score is expected to become a clinical tool for identifying the occurrence of HF in DM.

### 8.8 hs-cTnT and sST2

In a cohort study (66) that included 1069 patients with HF, the investigators categorized them into the HF complicated with DM group and the HF without DM group. The results showed that hs-cTnT and sST2 were the only markers independently associated with both all-cause and cardiovascular death in the DM-HF group, and the combination of the two significantly improved the predictive accuracy.

### 8.9 Molecular markers combined with imaging markers

Global Longitudinal Strain (GLS) is a quantitative measure of longitudinal contractile function of the LV myocardium assessed by cardiac ultrasound strain imaging, reflecting the overall shortening capacity of the myocardium in the long-axis direction (apical to basal).Flores-Ramírez et al. (68) found that GLS in combination with serum Gal-3 testing could noninvasively screen for subclinical LVDD in patients with asymptomatic DM, with patients with preserved EF having significantly higher Gal-3 levels and lower GLS values compared with patients with DM alone. Rørth et al. (69) followed up 960 T1DM patients with normal EF for 6.3 years, and found that echocardiographic E/E’ ratios in conjunction with serum NT-proBNP levels identified a population with very high cardiovascular risk (NT-proBNP >300 pg/mL and E/e′>12) and may provide a low-cost, easily accessible tool for accurate clinical stratification.

## 9 Conclusion and future perspectives

The European Society of Cardiology (ESC) HF guidelines (72) state that there is a significant evidence gap in the study of the pathogenesis of DM complicated with HF in the population, and there is currently no consensus in the academic community on the etiologic definition of DCM, resulting in a lack of specific clinical diagnostic criteria. Given the high morbidity and mortality of DCM, it is especially critical to establish an accurate early diagnosis system. Early identification can not only accurately assess the myocardial pathological status, but also delay the progression of heart failure through interventions. Recent studies have shown that circulating biomarkers (e.g., sST2, CT-1, galectin-3) correlate well with the pathologic stage of DCM, and their convenience and affordability make them potential diagnostic tools. It is worth noting that combined detection strategies (e.g., LOXL2 + ETFβ, LPL + ANGPTL4) can significantly improve diagnostic specificity, but face limitations such as long testing periods and high costs. Emerging markers such as EDN1 gene methylation and AF provide new ideas for non-invasive diagnosis, but their clinical translation still requires multi-center validation. The current core challenge in the field is: how to translate research results into clinical practice, focusing on when to implement, who will implement, and how to implement. In summary, the search for effective and implementable molecular markers has remained one of the key tasks in the field of DCM for decades.
